# Necrotizing fasciitis in a plaster casted upper extremity: a forensic autopsy case report

**DOI:** 10.1007/s00414-025-03548-5

**Published:** 2025-07-21

**Authors:** Ahed Alghaithi, Marie Epain, Anne-sohphie Advenier, Laurent Fanton

**Affiliations:** 1 Hospices civils de Lyon, Lyon, France; 2 Université Claude Bernard Lyon 1, Lyon, France

**Keywords:** Necrotizing fasciitis, Tissue necrosis, Cast, Plaster, *Streptococcus pyogenes* infection, *Staphylococcus aureus* infection

## Abstract

Necrotizing fasciitis (NF) is a serious life-threatening condition characterized by rapidly progressing tissue necrosis. It is associated with a high mortality rate, reaching up to 80% in some cases. Few cases of necrotizing fasciitis as a complication of casting have been reported, and most of these occurred in the orthopedic setting. The present article reports the case of a patient who developed necrotizing fasciitis following the placement of a cast and who was undiagnosed clinically. Due to the aggressive nature of the infection, the patient died shortly after the initial complaint and the case was presented to the forensic team for further evaluation by autopsy. Although cases of necrotizing fasciitis following cast application have been published in the literatures, no cases have been described within the forensic medicine setting. This is the first autopsy case report discussing this condition in a forensic medicine context, underscoring the novelty of this report. Our objective is to emphasize the importance of an early recognition of this devastating infection to avoid undesirable outcomes. Necrotizing fasciitis needs to be considered in patients with casts, particularly when presenting with pain out of proportion compared to the physical findings. The detailed autopsy findings along with the pathological and microbiological findings are also highlighted here to help forensic doctors and pathologists recognize this type of infection when encountered.

## Introduction

Necrotizing fasciitis (NF) is a serious life-threatening condition that can be defined as a severe, devastating inflammatory infection of the fascia, characterized by rapidly progressing tissue necrosis. It is associated with high mortality rate, reaching up to 80% in some cases. It is not only a rare condition with high mortality rate but it is also considered as a challenging case in terms of diagnosis and management [1]. From a pathophysiological point of view, the pathological agent in the majority of NF cases is introduced to the body through a break down in the skin integrity ranging from a minor trauma or procedure to more serious large wounds, open fractures, invasive procedures, or surgeries. The fact that there is less blood flow to the fascia is sufficient to create a good environment for the proliferation of the pathological agent that produces endotoxins and exotoxins leading to microvascular thrombosis. The end result of this process is poor microcirculation leading to tissue ischemia, cell death, and necrosis [1, 2]. Immunosuppression, diabetes mellitus, peripheral vascular diseases, chronic kidney diseases, obesity, smoking, and advanced age are all considered as a predisposing factors for NF. In addition, recent trauma and burns can increase the risk of acquiring such infections [3].

Microorganisms involved in NF include gram positive bacteria (e.g. *Staphylococcus aureus*, *Streptococcus pyogenes* and *Enterococci)*, gram negative bacteria (e.g. *Escherichia coli*, *Pseudomonas* species), and anaerobic organisms (*Bacteroides* species and *Clostridioides*) [4]. NF is classified into 4 types. Type I, which is the polymicrobial type, is reported to affect the truck and perineum and considered as the most common form, accounting for up to 70–80% of the reported cases; it mostly affects patients with diabetes mellitus or peripheral vascular disease. Type II, the monomicrobial type, is seen in 20% of the cases and has been mostly reported to affect the extremities. Type III, which is caused by gram negative bacteria and type IV, which is caused by fungus, are both considered as rare forms of NF [5].

Patients with early NF may present with severe pain, which can be disproportionate when compared to the physical findings, representing one of the classical manifestations of NF. Clinically, the patients can also present with a wide variety of signs ranging from skin edema and erythema to more serious signs like fever, tachycardia, and altered mental status [6]. Although the diagnosis of NF is mainly clinical, there are certain laboratory values and imaging studies that can aid in making and confirming the diagnosis in suspected cases. On plain radiography, the appearance of subcutaneous air is specific to NF but has low sensitivity. Although magnetic resonance imaging (MRI) is known for its high sensitivity in identifying fascia necrosis, there is no available data comparing its sensitivity and specificity to that of computed tomography (CT). However, since the diagnosis can be clinically-based, the use of these diagnostic tools should not delay the diagnosis and treatment, especially in critically ill patients. The Laboratory Risk Indicator for Necrotizing Fasciitis Score System (LRINEC) is recognized as a reliable diagnostic tool that has proven its effectiveness in differentiating NF from other soft tissue infections [7, 8]. The best prognostic outcomes are seen in cases where the diagnosis of NF is made early and followed by early and aggressive surgical debridement combined with the right antibiotic therapy. Conversely, in cases where the disease has been misdiagnosed as other non-necrotizing infections, such as abscess or cellulitis, the spread of the infection can be rapid and lead to undesirable outcomes such as amputation or death.

Although serious infectious complications following the placement of a cast or splint are considered to be rare, there are relatively few publications specifically related to NF as a complication of plaster casting. According to the orthopedic literature, certain groups of patients are considered to be at higher risk of developing such complications compared to others. These include patients with communication problems (such as those with extreme age, altered mental status, developmental delay), patients with hypoesthesia (neuropathy, nerve block), immunocompromised patients (diabetes mellitus, peripheral vascular diseases), and patients with spastic or cerebral palsy [3]. When reviewing the published cases of NF, 3 case reports of patients who developed necrotizing infections following the orthopedic application of a cast or splint were identified. To the best of our knowledge, this article is the first autopsy case report of a patient with NF related to a cast/plaster, in which the detailed autopsy findings along with the pathological and microbiological findings are highlighted to help forensic doctors recognize this type of infection when encountered.

## Case report

We report the case of a 49-year-old male, not known to have any medical comorbidities but with a history of regular alcohol consumption. He had a history of work accident in which his left wrist was injured. As per the police special report, it was a minor trauma after which he presented to his family doctor with a complaint of pain in his left arm. Left upper limb x-ray was performed and, since the result was insignificant, the patient was discharged on analgesia (paracetamol) and wrist support. Two days later, he presented again with a complaint of persistent pain in the left arm but no further investigation was undertaken; his wrist support was replaced by a plaster cast. The patient was discharged with the same analgesic drug and a follow-up plan in one week. The pain persisted and worsened, which made him remove the cast at home on day 9 after the injury. The same day he started to have fever, followed by confusion and cardiopulmonary arrest. Cardiopulmonary resuscitation was initiated by the family. Despite the intervention of the mobile emergency care service, the patient died.

**Fig. 1:**
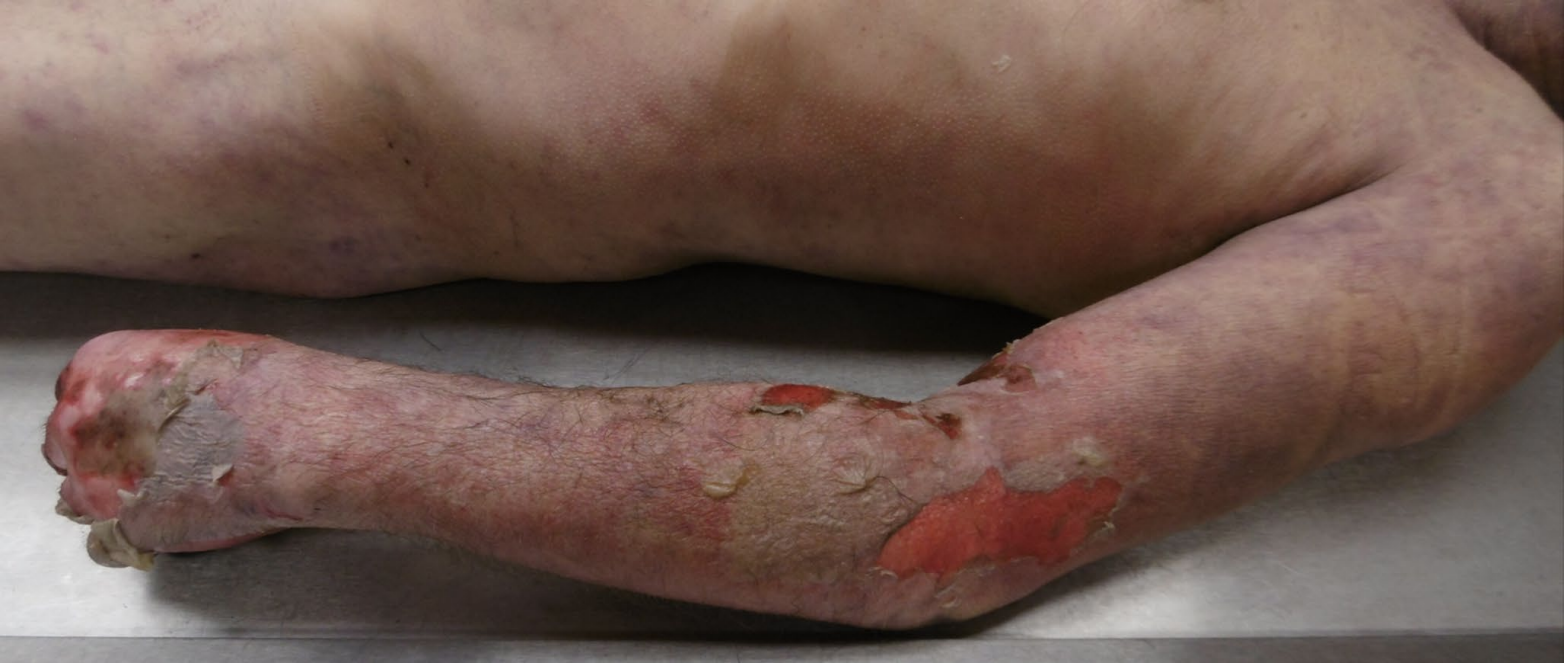
Multiple erythematous and purplish petechial zones along with skin detachments extending throughout the length of the left upper

In less than 24 h following the death, a forensic autopsy was performed to determine the cause of death and to rule out any medicolegal suspicion. External examination showed a male with a body mass index of 23.3 kg/m2. Multiple erythematous and purplish petechial zones along with skin detachments were found extending throughout the length of the left upper limb, covering the anterior surface of the arm up to the distal part of the forearm and the fingers (Fig. 1). The rest of the external examination showed no other significant findings.

**Fig. 2:**
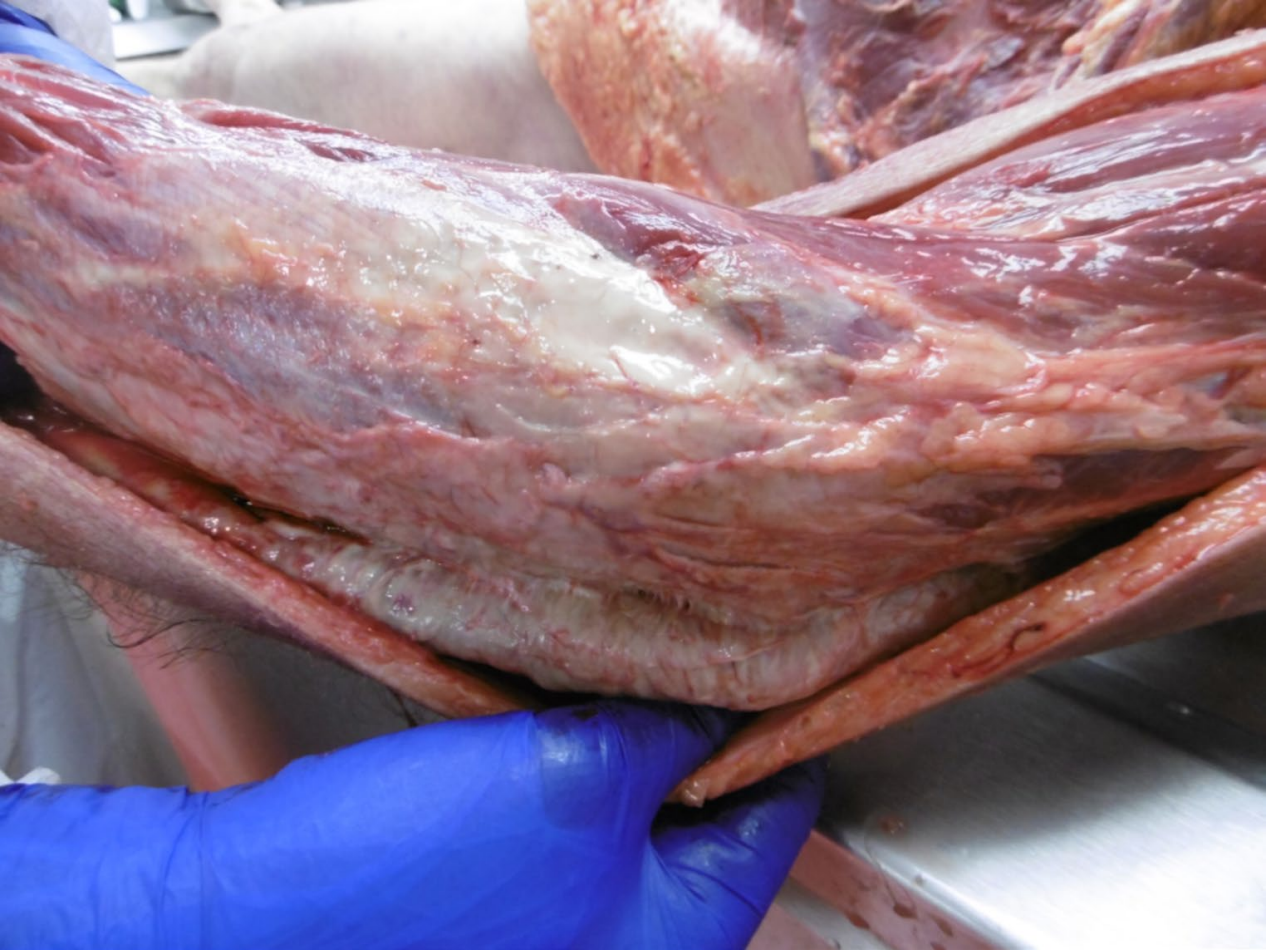
Evidence of purulent appearance at the level of the subcutaneous adipose tissue, the superficial and the deep fascia and the joint capsule

Upon internal examination, the dissection of the left upper limb showed evidence of purulent appearance at the level of the subcutaneous adipose tissue, the superficial and the deep fascia, and the elbow joint capsule (Fig. 2). At the level of the left hand and fingers, purulent and necrotic aspects of the subcutaneous tissue and the fascia surrounding the tendons were also observed. Elements suggestive of peritonitis were also noticed, including the presence of false membrane next to the small intestine and the left colic angle along with the presence of purulent discharge between the liver and the diaphragmatic dome. Upon opening the skull, congestion of the galea and the leptomeninges (arachnoid mater and pia mater), cerebral edema, and bilateral amygdala engagements were noted. The parenchyma of the lungs, liver, and spleen were moderately congested. The heart examination found atheromatous coronary artery disease mainly of the right coronary artery and the anterior descending artery, associated with an obstruction up to 40%. In the gastric mucosa, diffuse reddish petechiae and Wischnewski spots were present.

**Fig. 3:**
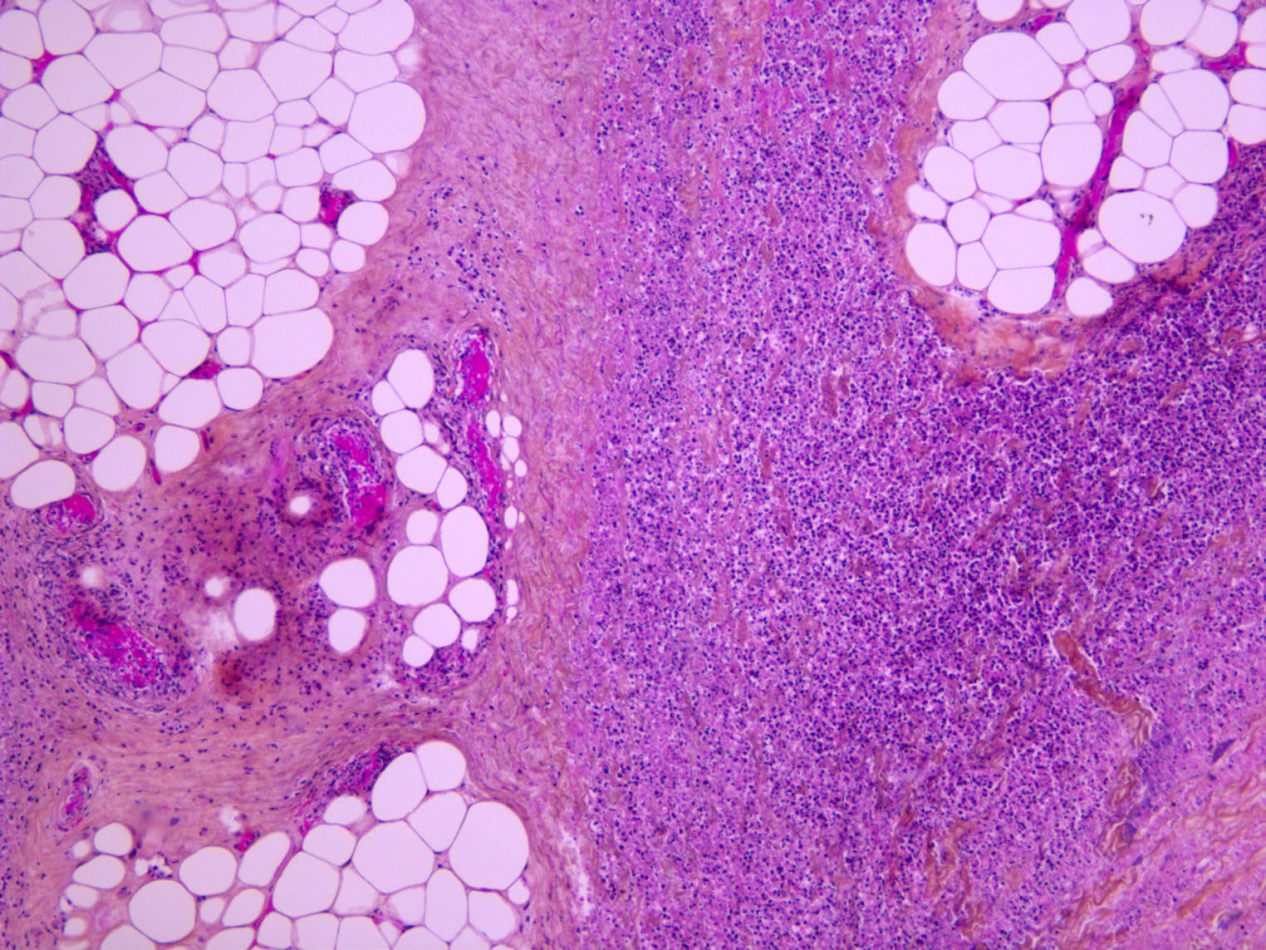
Acute necrotizing cellulitis of the left arm (HES x 100)

**Fig. 4:**
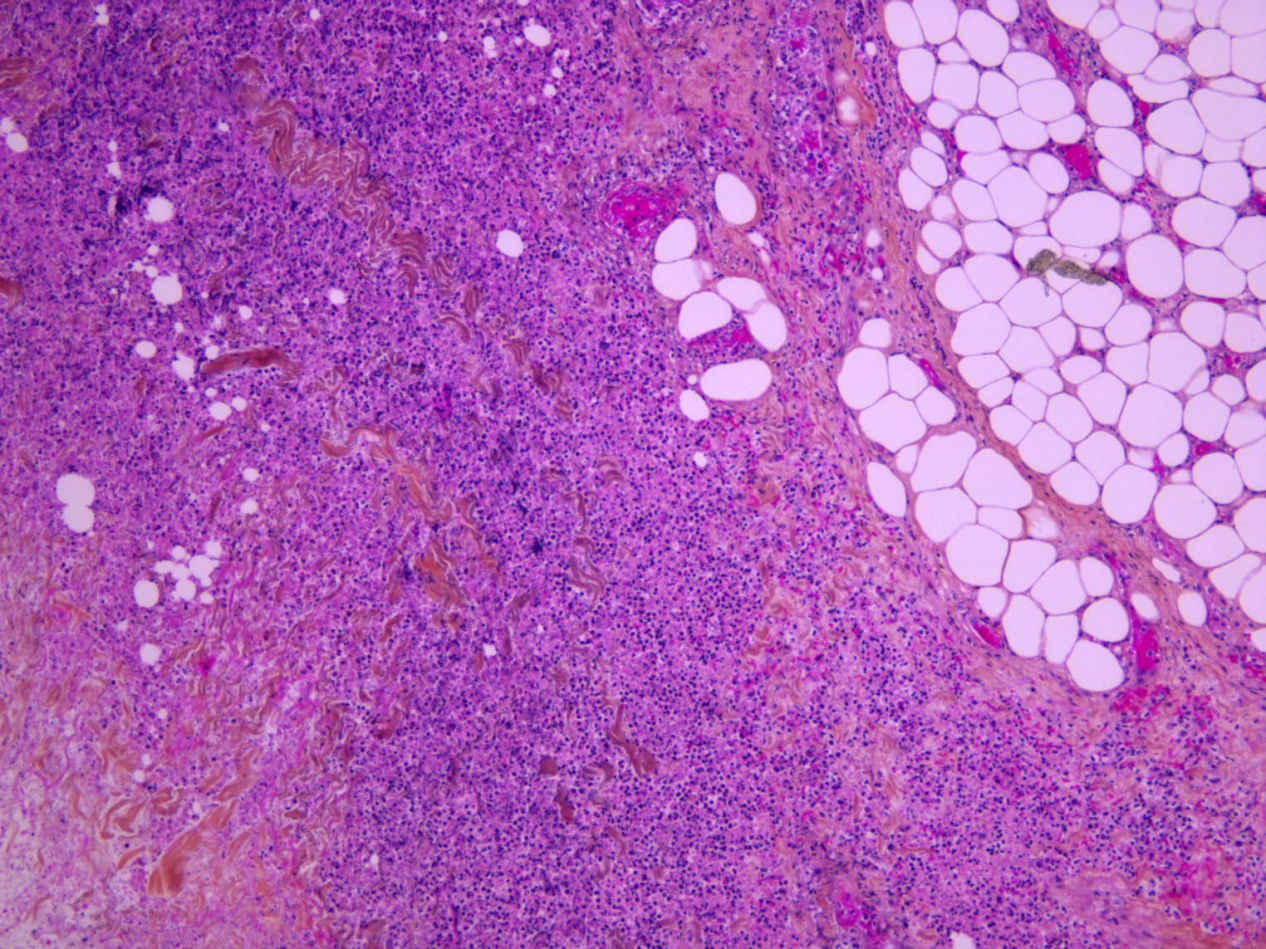
Acute necrotizing cellulitis of the left hand (HES x 100)

**Fig. 5:**
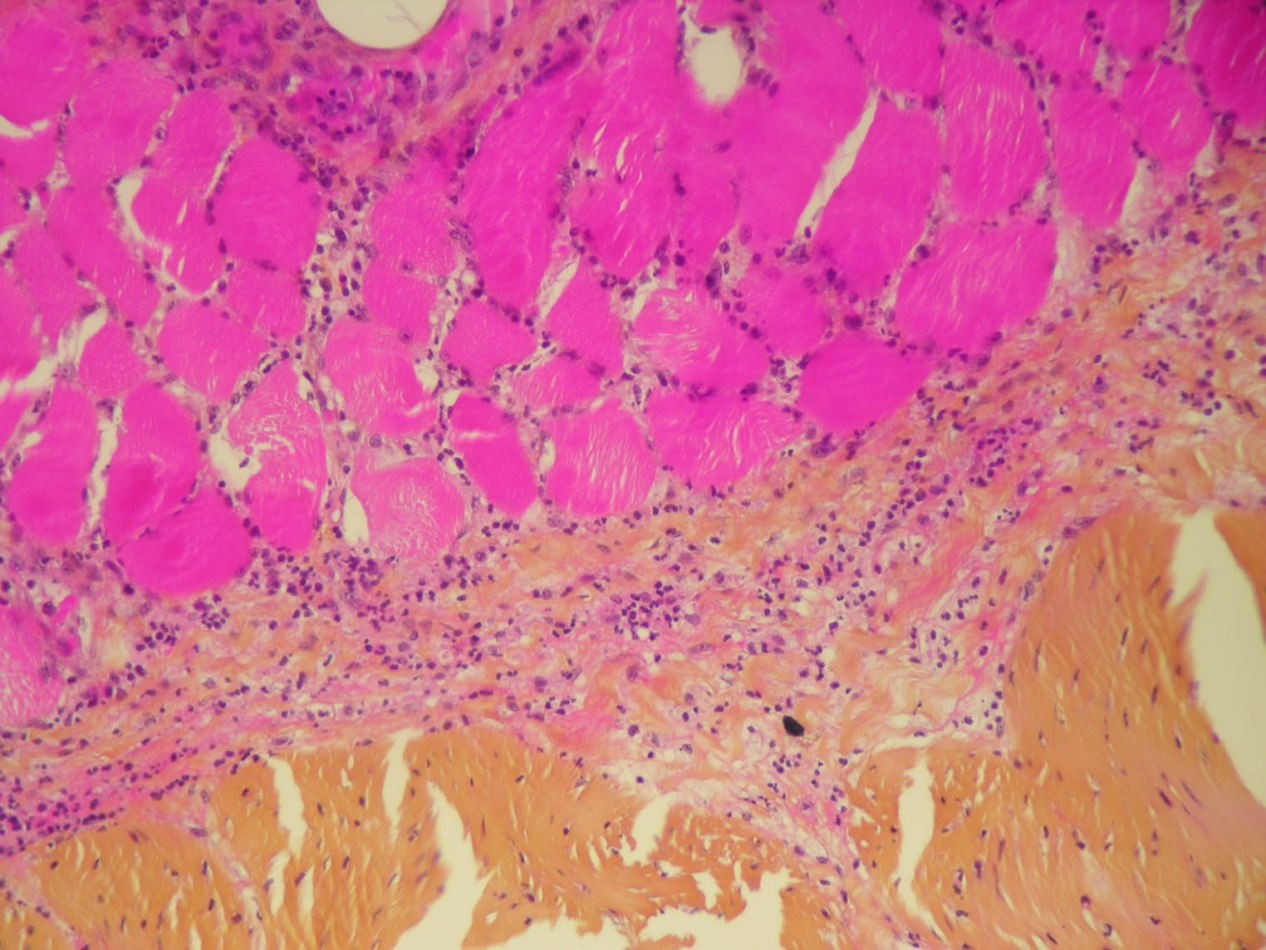
Subcutaneous and muscular infiltration by polymorphonuclear leukocytes (HES x 400)

**Fig. 6:**
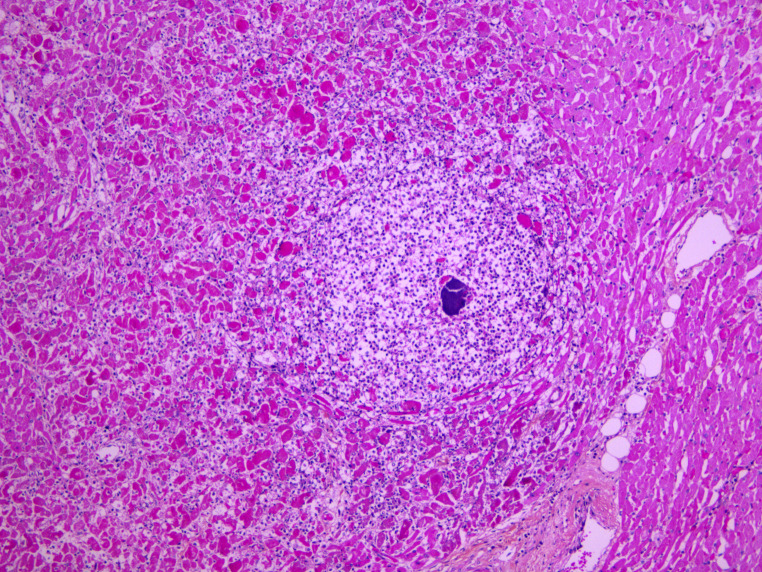
Acute myocarditis with micro-abscess (HES x 200)

**Fig. 7:**
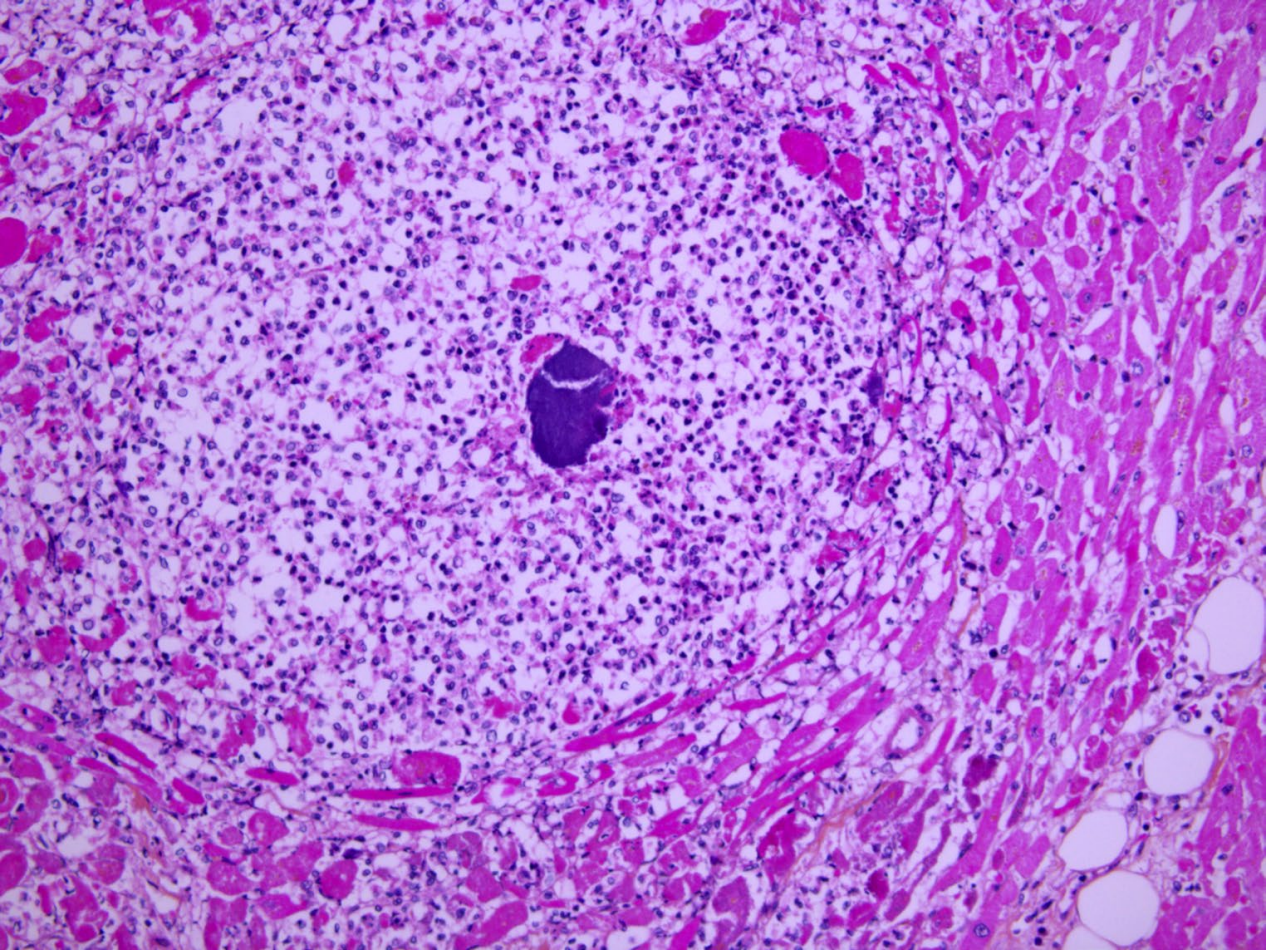
Micro-abscess with bacteria (HES x 400)

Standard histology staining (hematoxylin-eosin) was performed on all organs. It showed acute necrotizing cellulitis of the left upper limb dating approximately from 24 h before death (Figs. 3, 4 and 5). Additionally, peritonitis and acute myocarditis of the posterior and lateral left ventricular wall dating from more than 24 h before death were observed (Figs. 6 and 7). For the microbiological examination two blood samples were collected from the central venous system (CVS) for blood culture analysis. In addition, samples of the pericardial fluid, spleen biopsy, and upper limb muscle biopsy were also collected. *Streptococcus pyogenes* was found in all of the samples. *Staphylococcus aureus* was also found in the upper limb muscle biopsy, spleen biopsy, and the pericardial fluid.

Toxicological analysis was performed using central and peripheral blood samples along with a urine sample. A general unknown drug screening was carried out using gas chromatography/mass spectrometry and liquid chromatography/diode array detector/mass spectrometry. The concentration of detected molecules was measured by specific chromatographic techniques. Blood ethanol concentration was measured by gas chromatography/flame ionization detector. These analyses showed the presence of paracetamol 37.2 ug/ml (normal therapeutic blood level 5–25 ug/ml), codeine 124 ng/ml (30–250 ng/ml), and morphine analgesics 38 ng/ml (20–120 ng/L) at a blood concentration compatible with therapeutic doses. The presence of cannabinoids and its metabolites (THC, 11-OH-THC and THCCOOH ) was also detected at levels that could correspond to residues of chronic cannabinoid consumption, without excluding the possibility of a single isolated consumption several hours before death.

Biochemical analyses were also performed on the central and peripheral blood samples. The aim of these complementary tests was to measure the levels of CRP, NT-Pro BNP, and procalcitonin. The results showed a high concentration of these markers at 171 mg/L, 34,545 ng/L, and 1.14 ng/L in the central blood, respectively, and at 105 mg/L 14552ng/L, and 1.23ng/L in the peripheral blood, respectively.

In conclusion, from a forensic medicine point of view, after performing the detailed autopsy and the necessary examinations, we concluded that the cause of death in this case was acute generalized *streptococcus pyogens* infection secondary to a NF of the left upper limb, which was immobilized with a plaster cast for a few days.

## Discussion

In clinical practice, casting may be associated with serious life-threatening infectious and non-infectious complications, as shown in Table 1 [3]. Knowing the fact that casting may be associated with and might mask these complications, including NF as in the present case, highlights the importance of the clinical evaluation for patients with a cast or splint, especially those with clinical signs and symptoms pointing toward infection and sepsis. In such patients, the undesirable outcome such as amputation or death can be avoided by simply removing the cast/ splint, inspecting the affected area, performing the necessary throughout examination, establishing the diagnosis, and starting the treatment early [3].

**Table 1:** The infectious and non-infectious complications associated with casting

Infectious complications of casting/splinting	Non-infectious complications of casting/splinting
Cellulitis	Compartment syndrome
Abscess	Deep vein thrombosis
Necrotizing fasciitis	Pulmonary embolisms
Clostridium gangrenous	
Toxic shock syndrome	

In the present case, although the initial clinical presentation was not highly specific of NF, the history of recent work accident, even if described as a minor injury, the presence of a plaster cast, the persistence of pain, and the developing of fever should have raised the suspicion of an infectious complication of casting. Dissection revealed necrosis of the subcutaneous tissue as well as the superficial and the deep fascia, extending throughout the entire length of the left upper limb, including even the elbow joint capsule and the tendons. The pathological and microbiological examinations then confirmed this diagnosis.

While cases of NF following the application of casts have previously been published, none have been documented within the context of forensic medicine. Netzer and Fuchs reported a case of NF in a 43-year-old female who had undergone a close reduction and casting of a tibial fracture. The patient presented with general nonspecific symptoms such as malaise and weakness that rapidly progressed to more serious signs including tachypnea, hypotension, and altered mental status, after which the diagnosis of septic shock was made. Initially, the physician suspected and ruled out compartment syndrome. Later on, the removal of the cast, the detailed clinical examination, and the surgical consultation led to the diagnosis of NF. The authors emphasized on the necessity of an early diagnosis and intervention in cases with NF to improve the patient’s outcome [9].

Delasobera et al. presented two cases of serious infectious complications related to cast/splint in the pediatric age group. The first was of an 8-year-old girl who developed toxic shock syndrome as a complication of the fiberglass splint that was placed to manage her fractures of the distal radius and ulna. The second was a NF case resulting in upper extremity amputation in a 3-year-old child. The child had a splint that was placed following a Gartland type I (non-displaced) supracondylar fracture. He developed a gangrene of the right arm for which he underwent amputation of the affected arm along with debridement of the anterior chest wall due to the presence of extensive soft tissue necrosis [3].

In their publication “A Five Years Review of the Anaerobic, Necrotizing Soft Tissue Infections: A Nursing Perspective”, Skacel and Boyle reviewed a total of 25 patients with serious anaerobic NF that required serious medical or surgical intervention, along with ICU admission and nursing care. Two out of the 25 patients developed NF following an elective orthopedic procedure requiring the application of a cast [10].

The present case and the review of the medical literature of the topic points to several important conclusions. First, in similar clinical presentations, with the presence of plaster cast, it is crucial to keep in mind and eliminate all the infectious complications of splint/cast that can range from mild local skin infection to more serious and life-threatening complications, such as cellulitis, NF, toxic shock syndrome, and clostridium gangrenes. Second, the huge diversity in the clinical presentation and disease progression of NF emphasizes on the importance of having a high index of suspicion for NF along with an accurate diagnosis and early management in order to lower the mortality rate associated with this infection. Finally, cases of NF that are reported in forensic pathology settings are very limited; to our knowledge, this is the first reported autopsy case of NF related to casting. Nevertheless, NF should be considered by forensic medicine specialists and pathologists when performing the autopsy of a patient following trauma or casting. In such cases, they should be aware of all the findings referring to NF including the external examination and dissection findings as well as the relevant microbiological and histopathological analyses that need to be performed to confirm the diagnosis. This comprehensive understanding of NF can increase the accuracy of determining the cause of death, especially since it can be encountered in cases of sudden unexpected death or medical malpractice cases.

## Data Availability

Not applicable. Data sharing is not applicable to this article.
